# Multidisciplinary perspectives on pediatric nurse prescribing: a mixed-methods analysis of attitudes and consensus

**DOI:** 10.3389/fped.2026.1747318

**Published:** 2026-05-20

**Authors:** Juan Wang, Yajun Yue

**Affiliations:** 1 Nursing College of Shanxi Medical University, Fenyang, China; 2 Fenyang Hospital of Shanxi Province, Fenyang, China; 3 Nursing Department, Shouyang County Medical Group, Jinzhong, China

**Keywords:** attitude of health personnel, health policy, mixed methods, nurse prescribers, pediatric nursing, risk assessment

## Abstract

**Background:**

In China, expanding nursing roles to include nurse prescribing has been proposed as a potential strategy to improve the efficiency of pediatric healthcare. Its implementation requires a clear understanding of how pediatric nurses and physicians view medication safety, professional boundaries, and implementation conditions in child health.

**Methods:**

A convergent mixed-methods study was conducted among pediatric healthcare professionals from four tertiary hospitals in China. Quantitative data were collected using a validated questionnaire assessing attitudes, perceived benefits, risk perception, collaborative trust, training needs, and support for prescribing scope. Data were analyzed using comparative statistics and logistic regression. Qualitative responses to open-ended questions were examined thematically, and the quantitative and qualitative findings were integrated in a joint display matrix.

**Results:**

Nurses showed stronger support for nurse prescribing than physicians (4.32 ± 0.67 vs. 2.92 ± 0.75, *p* < 0.001). Multivariable analysis identified perceived benefits (OR = 2.08, 95% CI: 1.14–3.81, *p* = 0.018) and training needs (OR = 2.57, 95% CI: 1.07–6.16, *p* = 0.035) as significant positive predictors of support. Both groups agreed most strongly with prescribing for basic, low-risk medications. Integrated findings highlighted three implementation priorities: clear scope definition, standardized training, and legal-regulatory safeguards.

**Conclusions:**

Pediatric nurses and physicians differed in their overall attitudes, but their shared acceptance of low-risk prescribing suggests a feasible implementation pathway. The findings support a phased model of pediatric nurse prescribing built on interdisciplinary collaboration, structured training, and appropriate regulatory safeguards.

## Graphical Abstract

**Figure:**
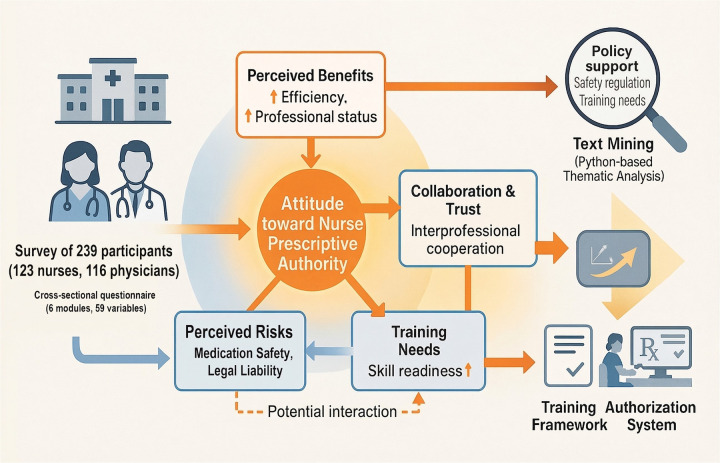
Multidimensional Perceptual and Policy Support Model Underpinning Pediatric Nurses’ Attitudes Toward Nurse Prescribing.

## Introduction

As healthcare systems place greater emphasis on integrated, multidisciplinary, and patient-centered care, nursing roles have continued to expand (1). In several countries, nurse prescribing has been introduced through independent or supplementary models and has been associated with improved access, service efficiency, and patient experience (2–4). Evidence also indicates that, within clearly defined scopes and training requirements, nurses can prescribe safely in selected primary care and chronic disease settings (5, 6). These international developments provide a relevant policy context for examining how nurse prescribing might be implemented in pediatric care.

In China, development of nurse prescribing remains at an early stage and is constrained by legal and regulatory boundaries that reserve prescribing authority for licensed physicians (2). Although local pilot discussions and protocol-based practices have emerged, they still lack unified national standards, stable legal support, and comprehensive regulatory oversight (7). As a result, concerns about legal liability, professional boundaries, and clinical competence continue to shape attitudes toward role expansion (8, 9). Existing Chinese studies have mainly described general attitudes or policy debates, whereas evidence remains limited on specialty-specific support, influencing factors, and implementation conditions. This study extends previous work by focusing on pediatric settings, comparing nurses and physicians directly, and integrating quantitative and qualitative evidence on feasibility and safeguards (10).

Pediatric nursing is characterized by a distinctive patient population, complex caregiving demands, and time-sensitive clinical decision-making, all of which place high demands on nurses' judgment and responsiveness (11). In routine practice, pediatric nurses are closely involved in medication-related tasks such as dose verification, monitoring, therapeutic feedback, and communication with caregivers. These responsibilities suggest that, under appropriate training and institutional safeguards, limited nurse prescribing could improve workflow and care responsiveness in pediatric settings (5). At the same time, pediatric pharmacotherapy carries heightened safety concerns because drug selection and dosing are especially sensitive in children. Empirical evidence on nurse prescribing in pediatric settings remains limited, which underscores the need to examine implementation feasibility, professional acceptance, and perceived safeguards in this specialty (12).

Against this background, this study examined support for nurse prescribing in pediatric care, the perceptions underlying that support, and the institutional barriers identified by frontline professionals. Specifically, it compared nurses and physicians, identified factors associated with support, and integrated survey findings with open-ended responses to clarify feasible implementation conditions. The study was guided by a multidimensional framework in which support for nurse prescribing was shaped by perceived benefits, perceived risks, collaborative trust, and training readiness. In this framework, attitude refers to overall evaluative orientation, perceptions refer to structured views of benefits, risks, and implementation conditions, and support refers to acceptance of policy implementation.

## Materials and methods

### Participants

This study used a convergent mixed-methods cross-sectional design so that quantitative patterns and qualitative implementation concerns could be collected during the same period, analyzed separately, and then compared directly at interpretation. Convenience sampling was adopted for feasibility and access to frontline staff, although this approach may limit representativeness and is considered in the Limitations section. The study was conducted between March and December 2024 in four tertiary general hospitals in Lvliang, Shanxi Province, China. Eligible pediatric nurses and physicians working in outpatient clinics, inpatient wards, or emergency departments were invited voluntarily through departmental coordination; recruitment was invitation-based rather than consecutive. Participants were classified as the nurse group and the physician group for analysis. The inclusion criteria were as follows: (1) age ≥22 years; (2) possession of a valid professional qualification certificate in the relevant field; (3) current employment as on-duty staff in pediatric outpatient clinics, inpatient wards, or emergency departments; (4) ability to communicate effectively with patients, families, and healthcare colleagues; and (5) informed consent and voluntary participation. The exclusion criteria were as follows: (1) less than 3 months of work experience in pediatric outpatient clinics, inpatient wards, or emergency departments; and (2) severe mental disorders, hearing impairment, or visual impairment that could affect comprehension or completion of the questionnaire. The study protocol was reviewed and approved by the hospital ethics committee (Approval No. 2024007), and all participants provided written informed consent.

### Sample size estimation

The primary outcome was the difference in support rates for pediatric nurse prescribing between the nurse group and the physician group. The sample size was calculated using the formula for comparing two independent proportions: *n* = [(Z*_α_*_/2_ + Z*_β_*)^2^ × (p_1_(1-p_1_) + p_2_(1-p_2_))]/(p_1_-p_2_)^2^, where p1 and p2 represent the expected support rates in the nurse and physician groups, respectively; Z*_α_*_/2_ corresponds to the critical value for a two-sided significance level of 0.05; and Z*_β_* corresponds to 80% power (13). The expected support rates of 65% among nurses and 45% among physicians were derived from our pilot survey, corresponding to an absolute difference of 20 percentage points. This difference was considered meaningful because it would indicate a practically relevant difference in implementation readiness, training needs, and policy support between the two professional groups (14). The required sample size was 96 participants per group. After allowing for approximately 15% invalid responses and bias correction, the final target sample size was set at 112 participants per group, with a minimum total sample size of 224 participants. The final sample of 239 participants therefore met the prespecified requirement and was adequate for the regression analyses performed.

### Survey questionnaire

This study adapted the questionnaire framework developed by Ling Donglan et al. (15) because it was one of the few instruments developed in the Chinese context to assess attitudes, perceived benefits, risks, collaboration, and training readiness related to nurse prescribing. The original framework was therefore appropriate conceptually and linguistically, but it required contextual adaptation for pediatric practice and for inclusion of both nurses and physicians. Based on the published framework, expert revision, and pilot testing, the questionnaire entitled Perceptions of Pediatric Healthcare Professionals on Nurse Prescribing was finalized. The questionnaire consisted of six modules and 59 variables, covering demographic characteristics and multidimensional perceptual measures (Supplementary Appendix S1, Supplementary Table S1).

The first section collected demographic information, including gender, age, educational level, professional title, years of experience, hospital grade, and department type. These variables were used to describe sample characteristics and assess intergroup comparability. The second section assessed participants' knowledge and attitudes toward nurse prescribing. It included items on overall attitudes (e.g., level of agreement and perceived professional implications), support for prescribing scope (covering 12 drug categories such as antimicrobials, respiratory, dermatological, and emergency medications), perceived benefits (9 items), risk perception (6 items), institutional trust and collaboration (4 items), training and competence (5 items), and three open-ended questions. Specifically, “training needs” referred to perceived needs for prescribing-related preparation, including pharmacology, legal and regulatory knowledge, supervised practice, and related clinical competencies. All items were rated on a five-point Likert scale (1 = strongly disagree, 5 = strongly agree). The questionnaire was administered in Chinese; because the source instrument and target respondents were both Chinese, no forward-back translation was required for data collection. The English wording presented in this manuscript was translated during manuscript preparation and checked by two bilingual researchers for semantic consistency.

The initial draft of the questionnaire was developed by the research team through a review of relevant domestic and international literature and policy documents. It was then evaluated by three nursing management experts and two pediatric clinical specialists, and a revised version was produced. This expert review supported content validity by confirming item relevance, clarity, and contextual suitability for pediatric settings. Before the formal survey, a pilot test was conducted among 20 healthcare professionals to assess language clarity, logical flow, and completion time. Based on the pilot feedback, further revisions were made and the final version was established. The pilot results showed good internal consistency (Cronbach's *α* = 0.864). In the full sample, internal consistency, the KMO statistic, and Bartlett's test were used as preliminary evidence that the multi-item constructs had acceptable internal coherence and structural adequacy for the planned analyses.

### Data collection and quality control

The formal survey was conducted between March and December 2024 using anonymous online questionnaires distributed through the Wenjuanxing platform. The target population included on-duty healthcare professionals working in pediatric outpatient clinics, inpatient wards, and emergency departments in four tertiary general hospitals. Eligible participants were invited through departmental coordination to complete the anonymous online questionnaire via QR codes or hyperlinks. An informed consent statement was displayed before participation, and respondents could access the questionnaire only after clicking “Agree and Start.” The effective response rate was calculated as the number of valid questionnaires divided by the number of distributed questionnaires multiplied by 100%. Because the survey was anonymous, detailed information on non-respondents was not available for comparison with respondents.

To ensure data quality and authenticity, several logic checks and control measures were embedded in the survey system: mandatory completion of all items, a minimum response time of 180 s, automatic blocking of duplicate IP addresses and device IDs, and consistency checks for response patterns, including reverse-coded items. After submission, all responses were reviewed and cleaned to remove invalid questionnaires with logical contradictions, missing critical items, or abnormally short completion times. Invalid responses were coded as “−99” and excluded from the primary analysis.

All data were exported to Excel for standardized coding and were independently verified by two researchers to ensure accuracy. The entire data collection process strictly complied with the Personal Information Protection Law and relevant ethical standards, ensuring the anonymity of all participants.

### Statistical methods

All data were analyzed using SPSS 26.0 and Python 3.11, with the pandas, scipy, and statsmodels packages. Raw questionnaire data were first checked for completeness and logical consistency, and only responses marked as valid (VALID = 1) were included in the analysis. Responses with missing key variables were excluded during data cleaning; therefore, no imputation procedure was required for the primary analyses. Based on the initial letter of the questionnaire ID (*N* for nurses and D for physicians), participants were classified into the nurse group and the physician group.

To assess the comparability between groups, demographic and occupational variables were first analyzed. Categorical variables (e.g., gender, education level, professional title, hospital grade, department type) were compared using the chi-square (*χ*2) test, with Cramer's V reported to indicate effect size. Continuous variables (e.g., age and years of experience) were analyzed using Welch's *t*-test because it is robust to unequal variances. Visual inspection and distributional checks indicated no severe departure from approximate normality for these variables. Effect sizes were interpreted using conventional thresholds: for Cohen's d, approximately 0.2, 0.5, and 0.8 represented small, medium, and large effects; for Cramer's V, approximately 0.1, 0.3, and 0.5 represented small, medium, and large effects. A two-tailed *p*-value < 0.05 was considered statistically significant. Because the item-level comparisons were exploratory and correlated, formal multiple-testing correction was not applied; instead, effect sizes and consistency of patterns across dimensions were emphasized in interpretation.

For the analysis of overall attitudes toward nurse prescribing (ATT1-ATT4), responses were measured on a five-point Likert scale ranging from 1 (strongly disagree) to 5 (strongly agree). Independent-samples t-tests were used to compare group means, and Cohen's d was calculated to quantify effect size. Items related to support for prescribing scope (CAT1-CAT12) and policy recommendations (POL4A-D) were analyzed using Welch's *t*-test for Likert-scale variables and chi-square tests for binary variables, with the corresponding effect sizes reported.

Perceived benefits (BEN1-BEN9) and risk perception (RISK1-RISK6) were analyzed using the same methods. In addition, reliability and preliminary structural adequacy were assessed. Internal consistency was evaluated using Cronbach's *α*, while the Kaiser-Meyer-Olkin (KMO) measure and Bartlett's test of sphericity were used to examine sampling adequacy and factorability. A Cronbach's *α* > 0.70 indicated acceptable reliability, while KMO >0.60 and Bartlett's test *p* < 0.05 suggested that the data were suitable for preliminary structural assessment.

For the analysis of influencing factors, the dependent variable was defined as support for nurse prescribing. Participants selecting “agree” or “strongly agree” on ATT1 (score ≥4) were coded as 1 to identify a clearly supportive position. This binary operationalization was adopted to facilitate interpretation in logistic regression and to distinguish affirmative support from neutral or negative responses, while the broader attitudinal variation was retained in the descriptive and construct-level analyses. Independent variables were selected based on theoretical relevance, previous literature, and their representation of key perceptual dimensions in this study. These variables included demographic and occupational characteristics, namely gender, age, education level, professional title, and years of experience, as well as the mean scores of perceptual dimensions, including perceived benefits, risk perception, institutional trust, training readiness, and overall attitude. For regression modeling, multi-item Likert-scale constructs were summarized as mean scores. Because each construct represented an aggregate of several conceptually related items rather than a single ordinal response, the resulting composite scores were treated as approximately continuous variables. A hierarchical stepwise logistic regression model was then constructed. In the first step, demographic and occupational variables were entered. In the second step, perceptual and attitudinal dimensions were added. In the third step, the interaction between group identity and risk perception was examined. Reported regression indicators included the coefficient (*β*), standard error (SE), Wald *χ*2, *p*-value, odds ratio (OR), and 95% confidence interval (CI). Before modeling, multicollinearity, influential collinearity patterns, and overall model fit assumptions relevant to logistic regression were checked using variance inflation factors (VIF), cell-count inspection, the Hosmer-Lemeshow goodness-of-fit test, and discrimination metrics. Model fit was evaluated using the area under the receiver operating characteristic curve (AUC), Nagelkerke R2, and the Hosmer-Lemeshow goodness-of-fit test. All variables were retained after acceptable collinearity levels were confirmed.

To analyze healthcare professionals' subjective views and policy recommendations regarding nurse prescribing, text mining and thematic analysis were performed on responses to three open-ended questions (OPN1-OPN3). All textual responses were first exported as raw data files, and invalid or blank entries were removed after manual review. A custom Python 3.11 script was then used for text preprocessing and high-frequency keyword extraction. The procedure included three steps. First, Chinese word segmentation and cleaning were performed. Regular expressions were used to extract phrases containing 2–8 consecutive Chinese characters, while common function words and non-informative terms were excluded. Second, word frequency analysis was conducted. Keywords were identified separately for the three questions: “Primary barriers to implementing nurse prescribing” (OPN1), “Key measures to ensure safe implementation” (OPN2), and “Recommendations for future development” (OPN3). The top 20 high-frequency items for each question were retained. Automated preprocessing and keyword extraction were used only as supportive analytic tools, whereas thematic coding was predominantly inductive and finalized through manual review and team consensus. Third, semantic aggregation and translation were performed. Synonymous or semantically similar expressions were manually merged into broader themes. Unified thematic labels and coding decisions were reviewed and cross-checked by the research team to improve interpretive credibility and were subsequently translated into English with manual verification. Because interpretation of qualitative responses may have been influenced by the researchers' disciplinary backgrounds and prior assumptions, all thematic decisions were critically discussed within the research team to reduce individual bias.

Quantitative and qualitative data were integrated using a convergent mixed-methods strategy. The two strands were analyzed independently and then merged at the interpretation stage through three steps: (1) side-by-side comparison of quantitative dimensions and qualitative themes; (2) construction of a joint display matrix to align statistical results with representative implementation concerns and suggestions; and (3) generation of integrated meta-inferences regarding perceived barriers, safety concerns, and implementation needs. This integration procedure was used to identify areas of convergence, complementarity, and divergence across the two datasets. Continuous variables were reported as mean ± standard deviation (SD), and categorical variables were presented as frequencies and percentages. Analytical outputs and visualizations were generated and exported to Excel using Python scripts to ensure reproducibility and consistency.

## Results

### Participant enrollment and sample characteristics

**Figure 1:**
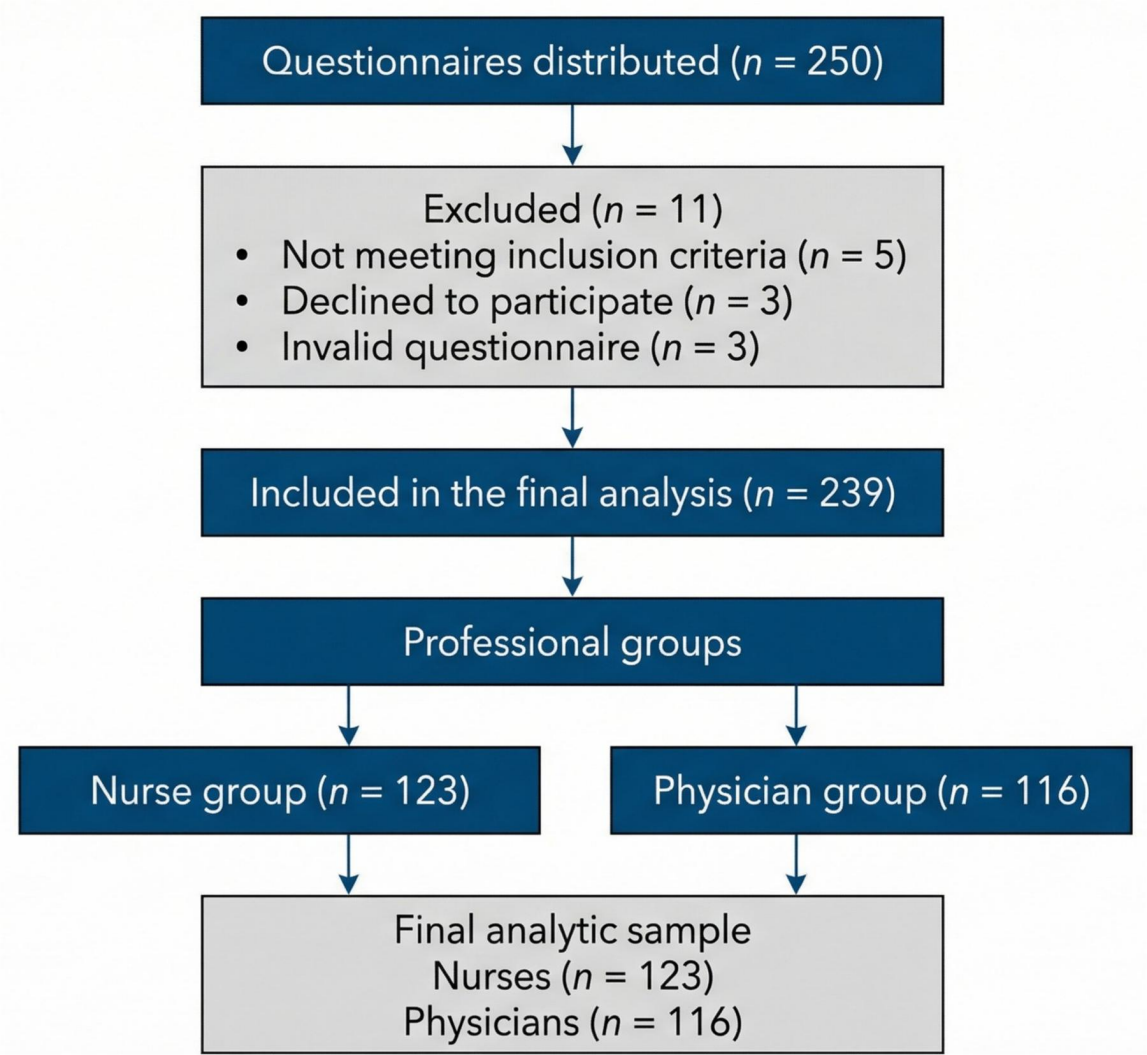
Flowchart of participant screening, exclusions, and final allocation of pediatric nurses and physicians. A total of 250 questionnaires were collected. Eleven were excluded due to invalidity (five did not meet the inclusion criteria, three declined participation, and three were deemed invalid), resulting in 239 valid questionnaires. Of these, 123 were assigned to the nurse group and 116 to the physician group, with no loss to follow-up or further exclusions.

A total of 250 questionnaires were distributed. After eligibility screening and data quality assessment, 239 valid questionnaires were included in the final analysis, yielding an effective response rate of 95.6% (239/250 × 100%). The final analytic sample consisted of 123 nurses and 116 physicians (Figure 1). Questionnaires with logical contradictions, missing critical items, or abnormally short completion times were considered invalid and excluded from the primary analysis.

### Comparison of general characteristics between the nurse and physician groups

**Figure 2:**
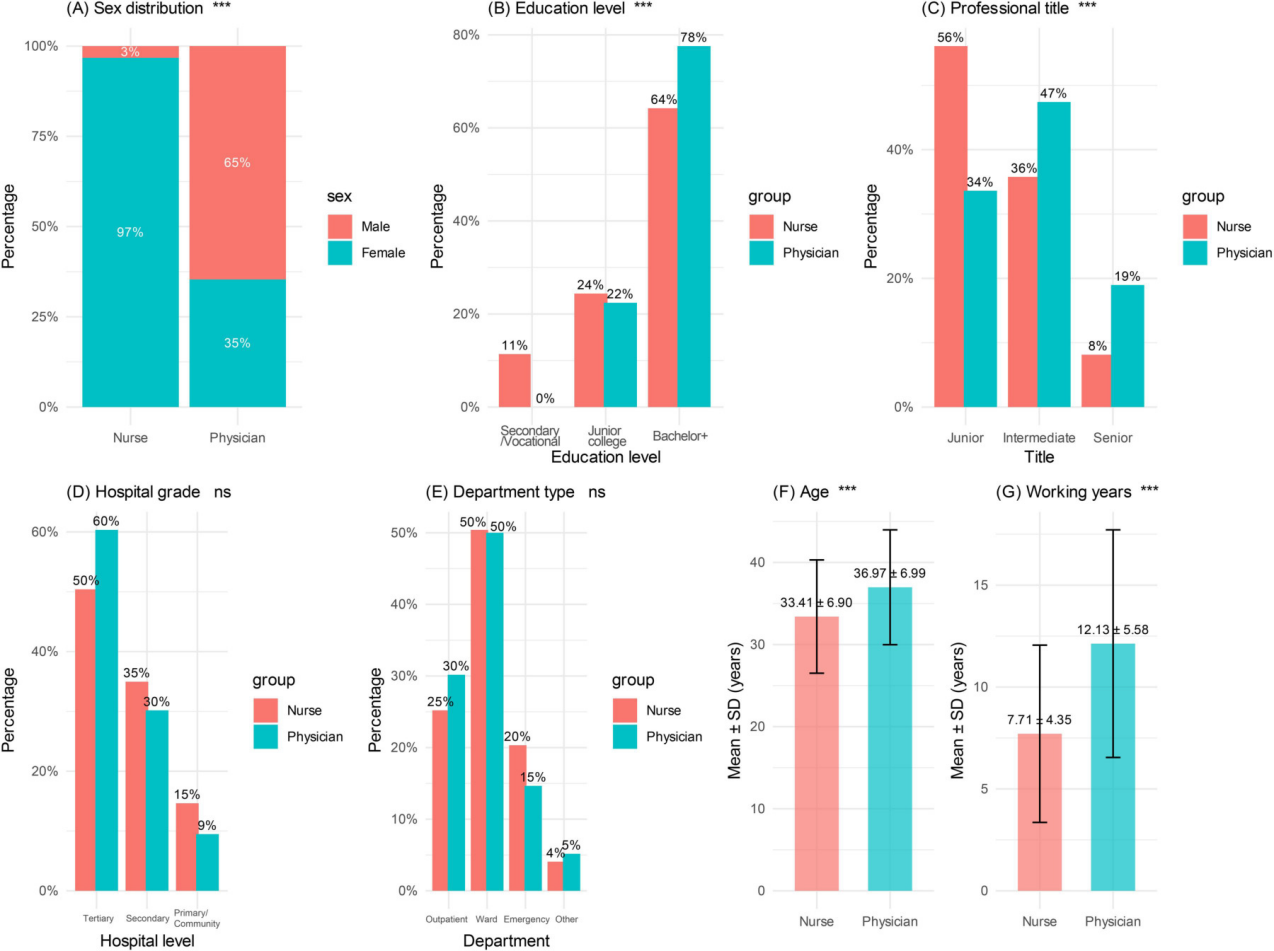
Comparison of demographic and professional characteristics between pediatric nurses and physicians. **(A)** Gender distribution; **(B)** Educational level; **(C)** Professional title; **(D)** Hospital grade; **(E)** Department type; **(F)** Age; **(G)** Years of experience. Each panel presents the distribution of demographic and occupational characteristics in the nurse and physician groups. The vertical axis indicates percentages or mean ± SD. Asterisks “*”, “**”, and “***” denote statistical significance at the levels of *p* *<* 0.05, *p* *<* 0.01, and *p* *<* 0.001, respectively. “ns” indicates no statistically significant difference.

To ensure comparability between the two groups, demographic and occupational characteristics were analyzed based on the 239 valid questionnaires collected (123 from the nurse group and 116 from the physician group). The results are presented in Table 1 and Figure 2.

**Table 1:** Comparison of general characteristics between nurse and physician groups (*n* = 239).

Variable	Category	Nurse Group *n* (%)	Physician Group *n* (%)	Test Statistic	*p*-value	Effect Size
Gender	Male/Female	4 (3.3)/119 (96.7)	75 (64.7)/41 (35.3)	*χ*^2^ = 98.961	<0.001	Cramer's *V* = 0.643
Highest Education	Secondary/Associate/Bachelor+	14 (11.4)/30 (24.4)/79 (64.2)	0 (0.0)/26 (22.4)/90 (77.6)	χ^2^ = 14.809	<0.001	Cramer's *V* = 0.249
Professional Title	Junior/Intermediate/Senior	69 (56.1)/44 (35.8)/10 (8.1)	39 (33.6)/55 (47.4)/22 (19.0)	χ^2^ = 13.862	<0.001	Cramer's *V* = 0.241
Hospital Level	Tertiary A/Secondary/Other	62 (50.4)/43 (35.0)/18 (14.6)	70 (60.3)/35 (30.2)/11 (9.5)	χ^2^ = 2.792	0.248	Cramer's *V* = 0.108
Department Type	Outpatient/Inpatient/Emergency/Other	31 (25.2)/62 (50.4)/25 (20.3)/5 (4.1)	35 (30.2)/58 (50.0)/17 (14.7)/6 (5.2)	χ^2^ = 1.787	0.618	Cramer's *V* = 0.086
Age (years)	—	33.41 ± 6.90	36.97 ± 6.99	*t* = −3.969	<0.001	Cohen's d = 0.514
Years of Experience	—	7.71 ± 4.35	12.13 ± 5.58	*t* = −6.801	<0.001	Cohen's d = 0.887

Data are presented as *n* (%) or mean ± standard deviation. Categorical variables were analyzed using Pearson's χ^2^ test, with Cramer's V reported as the effect size. Continuous variables were assessed using Welch's *t*-test, with Cohen's d reported as the effect size. Cramer's *V* > 0.5 indicates a strong effect, 0.3–0.5 a moderate effect, and <0.3 a weak effect.

The nurse group was predominantly female (96.7%), whereas the physician group had a significantly higher proportion of males (64.7%). This gender difference was highly significant (*χ*^2^ = 98.961, *p* *<* 0.001, Cramer's V = 0.643), indicating a strong effect size (Figure 2A). In terms of educational level, most nurses held a bachelor's degree or above (64.2%), while the corresponding proportion was higher among physicians (77.6%), and the difference between groups was statistically significant (*χ*^2^ = 14.809, *p* *<* 0.001, Cramer's *V* = 0.249) (Figure 2B). Regarding professional title, the nurse group was mainly composed of junior professionals (56.1%), whereas the physician group had a higher proportion of intermediate- or senior-level professionals (66.4%). This difference was also statistically significant (*χ*^2^ = 13.862, *p* *<* 0.001, Cramer's *V* = 0.241) (Figure 2C). No significant differences were observed in hospital grade (*p* *=* 0.248) or department type (*p* *=* 0.618), indicating good comparability of institutional level and departmental affiliation between the two groups (Figures 2D,E). The physician group had a significantly higher mean age (36.97 ± 6.99 years) than the nurse group (33.41 ± 6.90 years) (*t* = −3.969, *p* *<* 0.001, Cohen's d = 0.514), reflecting a moderate effect size. Physicians also had significantly more years of work experience (12.13 ± 5.58 years) compared to nurses (7.71 ± 4.35 years) (*t* = −6.801, *p* *<* 0.001, Cohen's d = 0.887), indicating a large effect size (Figures 2F,G).

In summary, the physician group showed significantly higher levels in educational attainment, professional title, age, and years of work experience, and the two groups also differed markedly in gender distribution. By contrast, no significant differences were found in hospital grade or department type. Because differences were observed in gender, age, educational level, professional title, and years of experience, these variables were retained in the descriptive comparisons to reflect the actual sample characteristics. In subsequent analyses of influencing factors, they were further included as covariates in the logistic regression models for sensitivity analysis and to test the robustness of the findings.

### Significant differences in overall attitudes toward nurse prescribing between nurses and physicians

**Figure 3:**
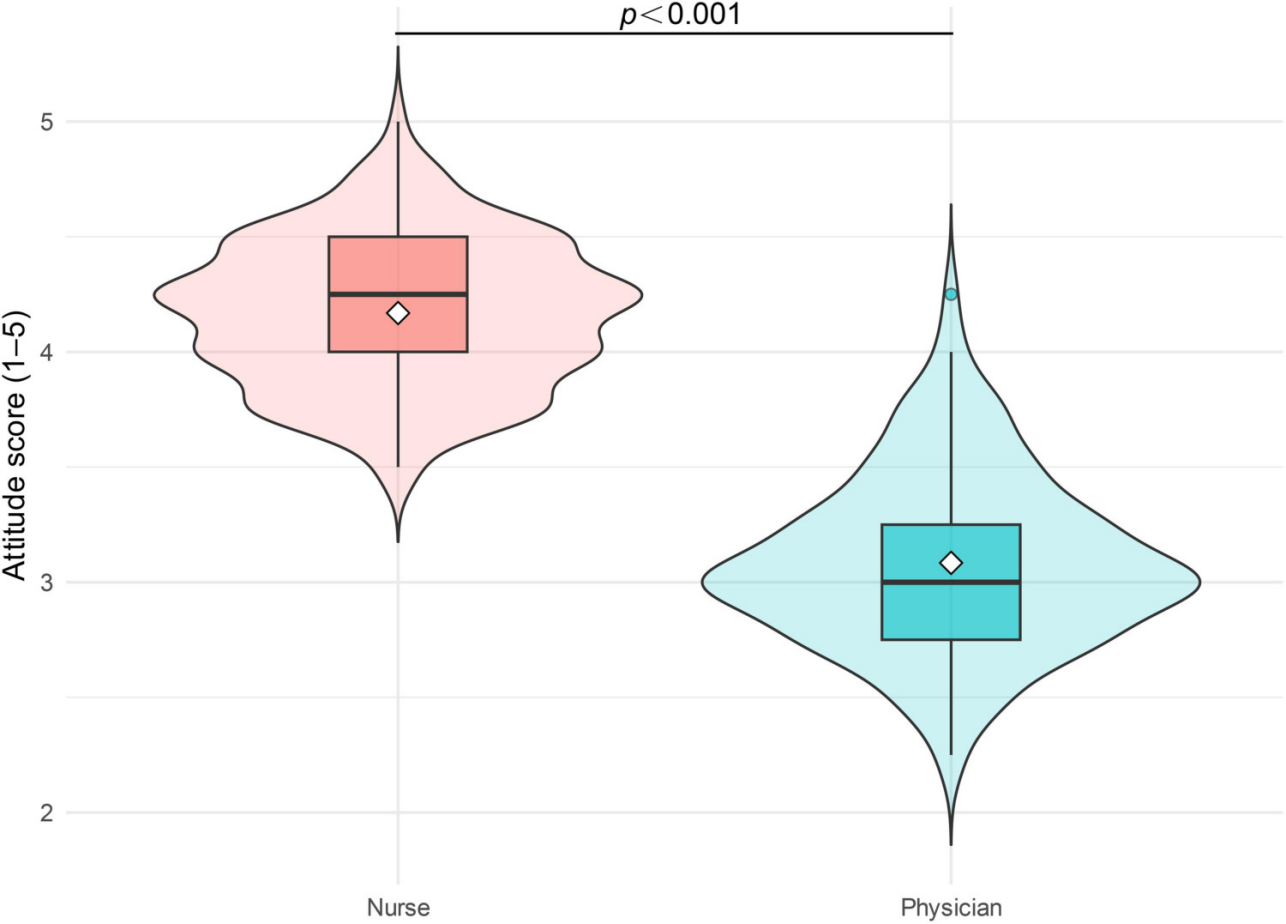
Comparison of overall attitudes toward nurse prescribing between nurses and physicians. Overall attitude scores were measured using a 5-point Likert scale (1 = strongly disagree, 5 = strongly agree). Violin plots illustrate the distribution density, while box plots represent the median and interquartile range (IQR), with whiskers extending to 1.5 × IQR. White diamonds (◇) indicate group means. The nurse group demonstrated significantly higher scores than the physician group (*t* = 15.162, *p* *<* 0.001, Cohen's d = 1.969).

The nurse group scored higher than the physician group across all attitudinal dimensions. For overall agreement, the mean score was 4.32 ± 0.67 among nurses and 2.92 ± 0.75 among physicians (*t* = 15.162, *p* < 0.001; Cohen's d = 1.969), indicating a large between-group difference (Figure 3).

As shown in Table 2, nurses expressed stronger agreement with statements such as “nurse prescribing can improve healthcare efficiency,” “it can reduce physicians’ workload,” and “it can elevate the professional status of nursing” (all *p* < 0.001). The corresponding effect sizes were all greater than 1.0, supporting the practical relevance of these differences. Overall, nurses were more likely to view nurse prescribing as a means of improving clinical workflow and strengthening the professional role of nursing, whereas physicians adopted a more cautious attitude.

**Table 2:** Comparison of overall attitudes toward nurse prescriptive authority between nurse and physician groups (*n* = 239).

Variable	Nurses (Mean ± SD)	Physicians (Mean ± SD)	*t*	*P*	Effect size (Cohen's d)
Overall agreement with nurse prescribing rights	4.32 ± 0.67	2.92 ± 0.75	15.162	<0.001	1.969
Prescriptive authority improves efficiency	4.21 ± 0.66	3.12 ± 0.71	12.295	<0.001	1.595
Prescriptive authority reduces physician workload	4.11 ± 0.67	3.19 ± 0.73	10.168	<0.001	1.32
Prescriptive authority enhances nursing professional status	4.03 ± 0.68	3.10 ± 0.89	9.055	<0.001	1.181

Data are presented as mean ± standard deviation. Independent-samples t-tests (Welch's correction) were performed. Effect sizes are reported as Cohen's d, where 0.2–0.5 indicates a small effect, 0.5–0.8 a medium effect, and ≥0.8 a large effect. All comparisons demonstrated large effect sizes, indicating substantial attitudinal differences between nurses and physicians.

### Nurses and physicians showed overall convergence in support for prescribing scope, with marginal differences on select items

**Figure 4:**
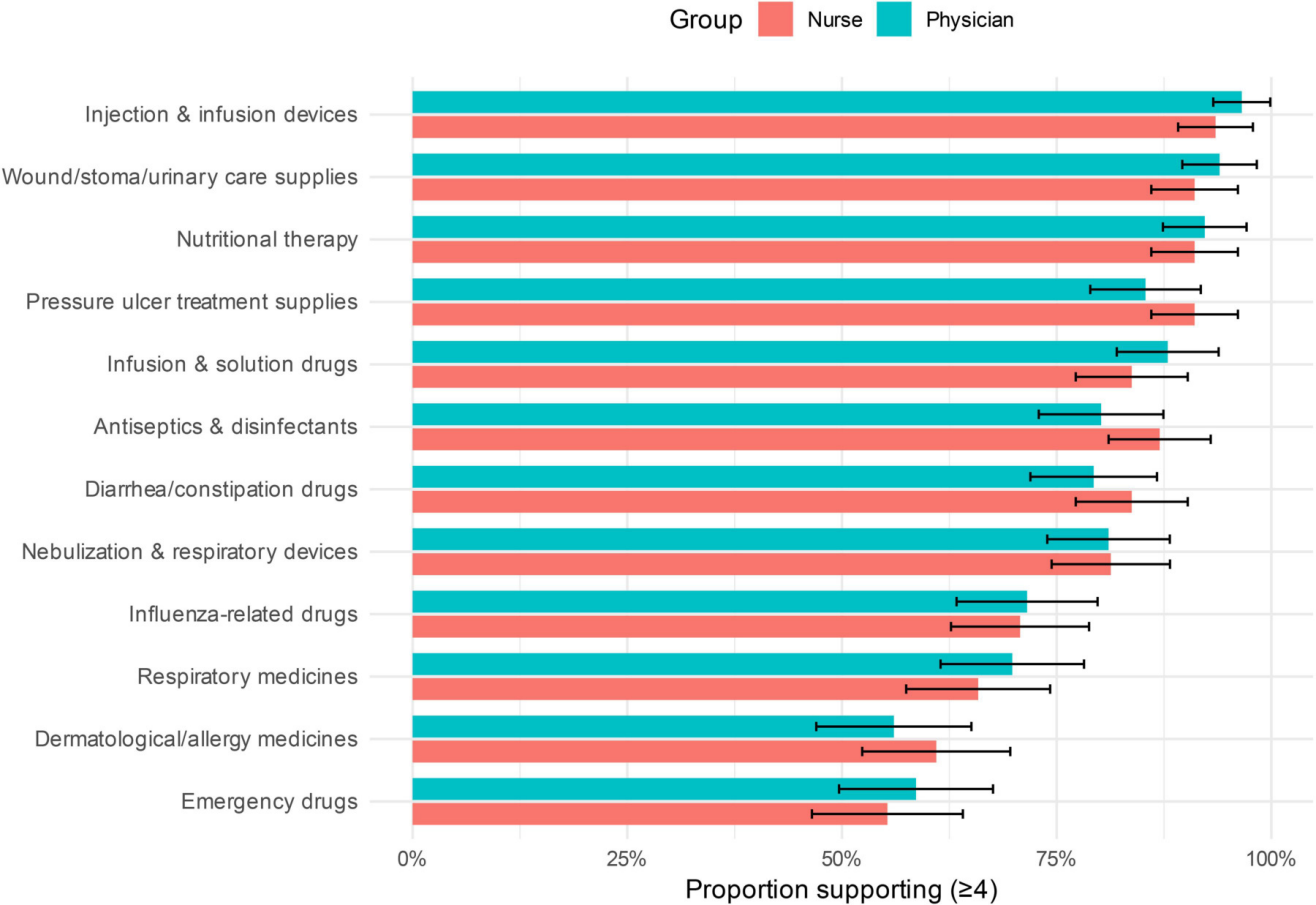
Group-specific support for nurse prescribing across 12 pediatric medication categories. The *x*-axis represents 12 categories of medications; the *y*-axis indicates the proportion of respondents expressing support (defined as a score ≥4). Bar height reflects support rate, and error bars denote 95% CIs. Group comparison: nurses vs. physicians. Support levels were calculated based on responses rated 4-5 on the Likert scale using valid questionnaires.

As shown in Figure 4 and Table 3, the two groups demonstrated generally similar levels of support across all 12 medication categories, with the strongest agreement concentrated on basic care and commonly used medications.

**Table 3:** Comparison of support levels for different prescription categories between nurses and physicians (*n* = 239).

Prescription category	Nurses (Mean ± SD)	Physicians (Mean ± SD)	t	P	Cohen's d
Antiseptics & disinfectants	3.98 ± 0.93	3.77 ± 1.08	1.653	0.1	0.215
Infusion & solution drugs	3.85 ± 0.95	3.96 ± 0.86	−0.884	0.378	−0.114
Respiratory medicines	3.54 ± 1.23	3.66 ± 1.16	−0.824	0.411	−0.106
Influenza-related drugs	3.64 ± 1.17	3.67 ± 1.19	−0.197	0.844	−0.026
Diarrhea/constipation drugs	3.98 ± 0.83	3.80 ± 1.17	1.313	0.191	0.172
Dermatological/allergy medicines	3.47 ± 1.26	3.21 ± 1.34	1.576	0.116	0.204
Nebulization & respiratory devices	3.89 ± 1.03	3.86 ± 1.16	0.169	0.866	0.022
Pressure ulcer treatment supplies	4.09 ± 0.74	4.04 ± 0.89	0.438	0.662	0.057
Injection & infusion devices	4.14 ± 0.76	4.22 ± 0.67	−0.926	0.356	−0.119
Wound/stoma/urinary care supplies	4.11 ± 0.81	4.22 ± 0.73	−1.018	0.31	−0.131
Nutritional therapy	4.07 ± 0.81	4.17 ± 0.75	−0.983	0.327	−0.127
Emergency drugs	3.28 ± 1.25	3.33 ± 1.24	−0.267	0.79	−0.035

Data are presented as mean ± standard deviation. Independent-samples *t*-tests (Welch's correction) were applied. Effect sizes are reported as Cohen's d (0.2–0.5 = small, 0.5–0.8 = medium, ≥0.8 = large). No statistically significant differences were observed between groups (*P* > 0.05), indicating similar perceptions of appropriate prescription scope.

Among all comparisons, only the item “disinfectants and antimicrobial agents” approached statistical significance, with nurses showing slightly greater support than physicians (nurse group: 3.98 ± 0.93; physician group: 3.77 ± 1.08; *t* = 1.653, *p* *=* 0.100, Cohen's d = 0.215), suggesting a more open attitude among nurses toward prescribing this class of basic drugs.

For all other categories, including solutions, respiratory medications, influenza treatments, medications for diarrhea or constipation, dermatological and allergy medications, nebulization and respiratory therapy devices, pressure injury supplies, injection and infusion equipment, wound, ostomy, and urinary care items, nutritional therapies, and emergency medications, no statistically significant differences were observed (all *p* > 0.05). All corresponding effect sizes were below 0.2, indicating negligible between-group differences.

Overall, nurses and physicians showed comparable levels of support across most medication categories, with broad agreement on nurses' potential role in prescribing basic and low-risk medications. At the same time, both groups remained cautious about extending prescribing authority to specialized or high-risk drugs. These findings suggest a high degree of consensus regarding prescribing scope and indicate a favorable basis for interprofessional collaboration.

### Differences between nurses and physicians in multidimensional perceptions of nurse prescribing

**Figure 5:**
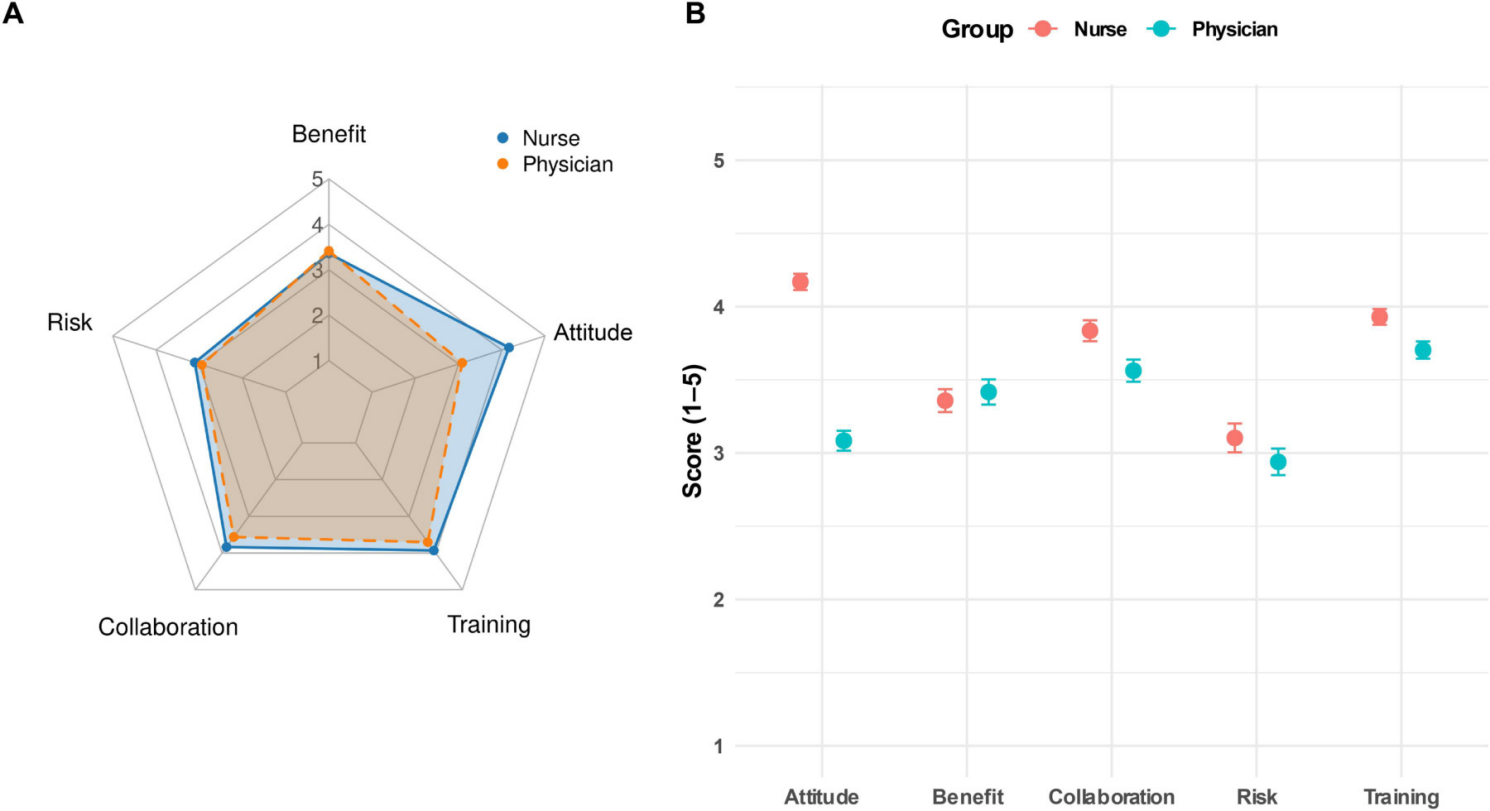
Comparison of perceptual dimensions related to nurse prescribing between nurses and physicians. **(A)** Radar chart illustrates the mean scores (1–5) for five perceptual dimensions: perceived benefits, risk perception, collaborative trust, training needs, and overall attitude; **(B)** Bar plot shows group means with 95% CIs, with significance indicated as *p *<* 0.05, **p *<* 0.01, ***p *<* 0.001. Dimension scores were calculated as the average of corresponding items using valid responses.

As shown in Table 4, nurses and physicians demonstrated broadly similar patterns across five dimensions related to nurse prescribing, including overall attitude, perceived benefits, risk perception, collaborative trust, and training needs. The clearest between-group difference was observed for overall attitude (*p* < 0.001) (Figure 5). Across all attitude items (ATT1-ATT4), nurses scored higher than physicians, with Cohen's d values ranging from 1.18 to 1.97, indicating consistently large group differences.

**Table 4:** Comparison of attitudes, perceived benefits, risks, collaboration, and training dimensions of nurse prescriptive authority between nurses and physicians and reliability/validity analysis of the scale (*n* = 239).

Domain	Item	Nurses (Mean ± SD)	Physicians (Mean ± SD)	*t*	*P*	Cohen's d
Attitude	ATT_1	4.32 ± 0.67	2.92 ± 0.75	15.162	<0.001	1.969
Attitude	ATT_2	4.21 ± 0.66	3.12 ± 0.71	12.295	<0.001	1.595
Attitude	ATT_3	4.11 ± 0.67	3.19 ± 0.73	10.168	<0.001	1.320
Attitude	ATT_4	4.03 ± 0.68	3.10 ± 0.89	9.055	<0.001	1.181
Benefit	BEN_1	3.69 ± 1.08	3.58 ± 1.25	0.748	0.455	0.097
Benefit	BEN_2	3.37 ± 1.29	3.43 ± 1.28	−0.343	0.732	−0.044
Benefit	BEN_3	3.37 ± 1.17	3.36 ± 1.27	0.024	0.981	0.003
Benefit	BEN_4	3.36 ± 1.28	3.56 ± 1.23	−1.250	0.213	−0.162
Benefit	BEN_5	3.32 ± 1.33	3.47 ± 1.17	−0.920	0.359	−0.119
Benefit	BEN_6	3.31 ± 1.29	3.41 ± 1.38	−0.556	0.579	−0.072
Benefit	BEN_7	3.53 ± 1.24	3.46 ± 1.23	0.448	0.655	0.058
Benefit	BEN_8	3.20 ± 1.35	3.36 ± 1.25	−0.992	0.322	−0.128
Benefit	BEN_9	3.08 ± 1.35	3.13 ± 1.32	−0.279	0.781	−0.036
Risk	RISK_1	3.00 ± 1.36	2.63 ± 1.37	2.099	0.037	0.272
Risk	RISK_2	3.20 ± 1.38	3.12 ± 1.23	0.441	0.660	0.057
Risk	RISK_3	3.42 ± 1.26	3.17 ± 1.32	1.497	0.136	0.194
Risk	RISK_4	2.89 ± 1.40	2.84 ± 1.36	0.277	0.782	0.036
Risk	RISK_5	2.82 ± 1.39	2.85 ± 1.27	−0.187	0.851	−0.024
Risk	RISK_6	3.28 ± 1.27	3.02 ± 1.32	1.594	0.112	0.207
Collaboration	COLL_1	3.60 ± 0.82	3.22 ± 0.92	3.420	<0.001	0.444
Collaboration	COLL_2	3.67 ± 0.76	3.41 ± 0.72	2.807	0.005	0.363
Collaboration	COLL_3	4.20 ± 0.65	4.16 ± 0.68	0.456	0.649	0.059
Collaboration	COLL_4	3.86 ± 0.81	3.47 ± 0.74	3.946	<0.001	0.509
Training	TRN_1	3.93 ± 0.66	3.73 ± 0.69	2.228	0.027	0.289
Training	TRN_2	3.79 ± 0.72	3.59 ± 0.75	2.047	0.042	0.265
Training	TRN_3	4.23 ± 0.66	4.07 ± 0.60	1.941	0.053	0.251
Training	TRN_4	3.75 ± 0.73	3.33 ± 0.66	4.683	<0.001	0.604
Training	TRN_5	3.96 ± 0.82	3.79 ± 0.84	1.544	0.124	0.200

Reliability and validity analysis: Cronbach's *α* = 0.862 (overall), KMO = 0.871, Bartlett's test of sphericity χ^2^ = 1,254.36, *P* < 0.001.

Data are presented as mean ± standard deviation. Independent-samples t-tests (Welch's correction) were used to compare groups.

Effect sizes are reported as Cohen's d, where 0.2–0.5 indicates a small effect, 0.5–0.8 a medium effect, and ≥0.8 a large effect.

Significant differences are marked in bold (*P* < 0.05). Reliability and validity indices indicate good internal consistency and structural stability of the measurement scale.

“Item” refers to the code of each questionnaire statement.

ATT = Attitude toward nurse prescribing; BEN = Perceived benefits;.

RISK = Perceived risks; COLL = Collaboration and institutional trust;.

TRN = Training and self-efficacy.

Each dimension score was calculated as the mean of its corresponding items (see Supplementary Table S1 for full item text).

In the dimension of perceived benefits, including items such as “improves the quality of nursing services,” “enhances work efficiency,” and “increases professional fulfillment,” the two groups reported similar mean scores ranging from 3.3 to 3.7. None of these differences reached statistical significance (*p* = 0.21–0.98), and all effect sizes were below 0.2, suggesting that both groups generally recognized the potential benefits of nurse prescribing (Figure 5A).

In the dimension of risk perception, which included items such as “medication-related risks,” “potential threats to patient safety,” and “risk of prescription errors,” physicians scored slightly higher than nurses, indicating greater concern about potential risks. However, only RISK1 showed a statistically significant difference (*p* = 0.037, Cohen's d = 0.27), whereas the remaining items were not significantly different between groups (Figure 5B).

In the dimensions of collaborative trust and training needs, several items reached statistical significance (*p* *<* 0.05). Specifically, nurses scored higher on COLL_1_, COLL_2,_ and COLL_4_, as well as on TRN_1_, TRN_2_, and TRN_4_, suggesting stronger agreement among nurses regarding the importance of institutional support, interprofessional collaboration, and training readiness.

Overall, the two groups showed broad consistency in their perceptions of the potential benefits and risks of nurse prescribing. However, nurses expressed a significantly more positive overall attitude and greater awareness of training needs, whereas physicians showed a somewhat more cautious perception of potential risks. Reliability and validity analyses demonstrated good psychometric performance of the scale across dimensions, with all Cronbach's *α* coefficients exceeding 0.85, a KMO value of 0.871, and a Bartlett's test of sphericity of *p* < 0.001. These findings support the structural stability, internal consistency, and applicability of the scale for assessing perceptions of nurse prescribing among healthcare professionals.

### Factors influencing support for nurse prescribing: multivariable logistic regression analysis

Support for nurse prescribing (defined as ATT1 ≥4 = 1; ≤3 = 0) was used as the dependent variable in both univariate and multivariate logistic regression models. The results are presented in Table 5. Univariate analysis showed that male gender, older age, higher education level, senior professional title, and longer years of experience were associated with lower support (*p* < 0.05). In contrast, higher scores for collaborative trust, training needs, and overall attitude were associated with greater support (*p* < 0.01). This pattern may partly reflect differences in sample composition, as the physician group included a higher proportion of males and individuals with advanced education and senior titles and generally showed a more cautious stance toward nurse prescribing.

**Table 5:** Logistic regression analysis of factors associated With support for nurse prescriptive authority (*n* = 239).

Predictor	Model 1 OR (95% CI), p	Model 2 OR (95% CI), p	Model 3 OR (95% CI), p
SEX_M (male = 1)	0.28 (0.15–0.53), <0.001	0.58 (0.28–1.19), 0.157	3.44 (1.06–11.23), 0.040
AGE (years)	0.97 (0.93–1.01), 0.179	0.98 (0.93–1.02), 0.353	1.00 (0.95–1.06), 0.930
EDU (higher=↑)	0.48 (0.27–0.83), 0.009	0.63 (0.33–1.18), 0.151	0.52 (0.24–1.14), 0.102
TITLE (higher=↑)	0.64 (0.42–0.98), 0.042	0.67 (0.40–1.11), 0.119	0.89 (0.50–1.59), 0.699
WORKY (years)	0.92 (0.87–0.98), 0.006	0.93 (0.87–0.99), 0.026	0.98 (0.91–1.05), 0.531
Benefit cognition (BEN_mean)	—	2.08 (1.14–3.81), 0.018	1.13 (0.48–2.65), 0.787
Risk cognition (RISK_mean)	—	0.75 (0.39–1.43), 0.383	0.41 (0.16–1.08), 0.071
Collaboration cognition (COLL_mean)	—	1.64 (0.85–3.17), 0.140	0.47 (0.19–1.21), 0.118
Training need (TRN_mean)	—	2.57 (1.07–6.16), 0.035	1.69 (0.50–5.66), 0.398
Overall attitude (ATT_mean)	—	3.34 (1.83–6.09), <0.001	0.79 (0.30–2.03), 0.619
NURSE (nurse = 1)	—	—	1.69 (0.02–172.08), 0.825
NURSE × RISK_mean	—	—	4.11 (0.92–18.29), 0.063

Model diagnostics.

Model 1: AUC = 0.780, Nagelkerke R^2^ = 0.289, Hosmer-Lemeshow *p* = 0.328.

Model 2: AUC = 0.846, Nagelkerke R^2^ = 0.443, Hosmer-Lemeshow *p* = 0.037.

Model 3: AUC = 0.905, Nagelkerke R^2^ = 0.621, Hosmer-Lemeshow *p* = 0.883.

Model 3 demonstrates the best fit and highest explanatory power. All predictor variables had VIF <2, indicating no evidence of multicollinearity.

The dependent variable is binary: support for nurse prescriptive authority (ATT_1≥4 = 1; ≤3 = 0).

Model 1 includes demographic and professional variables.

Model 2 adds cognitive variables (benefit cognition, risk cognition, collaboration cognition, training need, overall attitude).

Model 3 incorporates the interaction term “NURSE × RISK_mean.”.

The reference group comprises female participants with lower education, junior professional titles, and physician status. Continuous predictors were entered as raw scores.

The reversal in the direction of the gender effect in Model 3 results from the interaction term. Given the sample distribution—approximately 97% of nurses were female, and 65% of physicians were male—interpretation of the gender effect is conditional and does not reflect the overall trend.

In Multivariate Model 1 (including only demographic and occupational variables), gender, education level, professional title, and years of experience remained significant predictors. Male respondents (OR = 0.28, 95% CI: 0.15–0.53, *p* < 0.001) and those with more years of experience (OR = 0.92, 95% CI: 0.87–0.98, *p* = 0.006) were significantly less likely to support nurse prescribing, suggesting that baseline demographic characteristics independently influenced attitudes.

In Model 2, which incorporated perceptual variables in addition to demographic factors, the effects of the original covariates were markedly attenuated. Perceived benefits (OR = 2.08, 95% CI: 1.14–3.81, *p* = 0.018), training needs (OR = 2.57, 95% CI: 1.07–6.16, *p* = 0.035), and overall attitude (OR = 3.34, 95% CI: 1.83–6.09, *p* < 0.001) emerged as significant positive predictors. These findings suggest that perceptual and attitudinal factors play a central role in shaping healthcare professionals' positions on nurse prescribing.

After the interaction term between professional identity and risk perception was introduced in Model 3, gender remained statistically significant (OR = 3.44, 95% CI: 1.06–11.23, *p* = 0.040), although the direction of the coefficient changed relative to the baseline model. This pattern should be interpreted cautiously, as it may reflect coefficient instability after adjustment for additional model terms rather than a substantive reversal.

**Figure 6:**
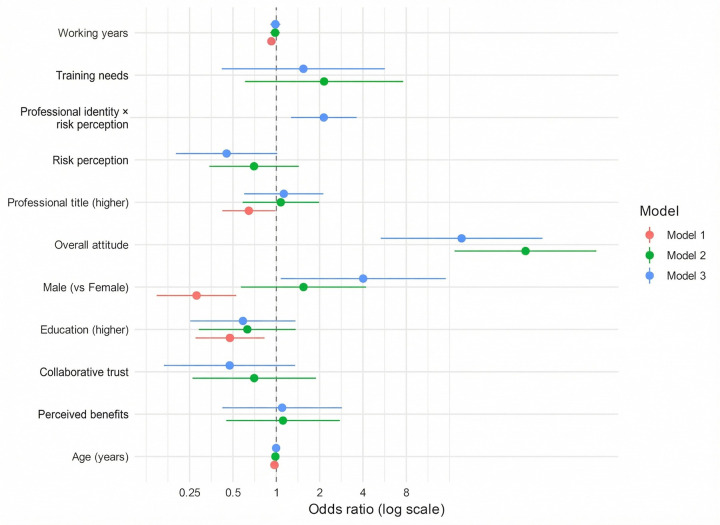
Factors associated with support for nurse prescribing (logistic regression analysis). The figure presents ORs and 95% CIs derived from logistic regression models. The *y*-axis lists independent variables, including demographic characteristics and perceptual dimensions; the *x*-axis displays ORs on a logarithmic scale. Models 1–3 represent: a baseline model with demographic variables, a model incorporating perceptual variables and a model including interaction terms. Analysis was based on respondents with complete data.

The interaction effect (NURSE × RISKmean) approached statistical significance (OR = 4.11, 95% CI: 0.92–18.29, *p* = 0.063), suggesting a possible difference in how risk perception related to support across professional groups. The model demonstrated good overall fit (AUC = 0.905, Nagelkerke R2 = 0.621, Hosmer-Lemeshow test *p* = 0.883), indicating strong explanatory performance (Figure 6).

Overall, support for nurse prescribing was associated with gender, professional identity, and perceptual factors. Greater perceived benefits, stronger training needs, and a more positive overall attitude were associated with stronger support. Although the pattern of risk perception appeared to differ between professional groups, the interaction between professional identity and risk perception did not reach statistical significance and should therefore be interpreted as exploratory.

### Thematic aggregation and conceptual synthesis of open-ended responses

The thematic framework of nurse prescribing centered on three core areas: institutional barriers, safety assurance, and future directions for development (Table 6).

**Table 6:** Key themes and frequencies From open-ended responses.

Variable	Item Description	Revised Keyword (EN)	Frequency	Percentage (%)
OPN_1	Primary barriers to implementing nurse prescriptive authority	Define prescription scope/criteria	36	7
OPN_1	Primary barriers to implementing nurse prescriptive authority	Entry and training standards	36	7
OPN_1	Primary barriers to implementing nurse prescriptive authority	Pilot programs	35	6.8
OPN_1	Primary barriers to implementing nurse prescriptive authority	Experienced nurses	30	5.9
OPN_1	Primary barriers to implementing nurse prescriptive authority	Gradual empowerment is the trend	30	5.9
OPN_1	Primary barriers to implementing nurse prescriptive authority	Oversight and regulatory mechanisms	30	5.9
OPN_1	Primary barriers to implementing nurse prescriptive authority	Efficiency gains from nurse prescribing	29	5.7
OPN_1	Primary barriers to implementing nurse prescriptive authority	Reduce physician workload	29	5.7
OPN_1	Primary barriers to implementing nurse prescriptive authority	Faster patient access to medicines	27	5.3
OPN_1	Primary barriers to implementing nurse prescriptive authority	Pharmacology knowledge gaps	20	3.9
OPN_1	Primary barriers to implementing nurse prescriptive authority	Knowledge	20	3.9
OPN_1	Primary barriers to implementing nurse prescriptive authority	Significant risks	20	3.9
OPN_1	Primary barriers to implementing nurse prescriptive authority	Unclear liability concerns	17	3.3
OPN_2	Key measures for safe implementation	Physician supervision/mentorship system	39	16.3
OPN_2	Key measures for safe implementation	Tiered prescribing authority	36	15.1
OPN_2	Key measures for safe implementation	Drug safety and traceability	35	14.6
OPN_2	Key measures for safe implementation	Strengthen training and assessment	29	12.1
OPN_2	Key measures for safe implementation	Lack of unified standards	20	8.4
OPN_2	Key measures for safe implementation	Legal risks	13	5.4
OPN_2	Key measures for safe implementation	Inadequate regulation	7	2.9
OPN_3	Recommendations for future development	Protect patient rights	36	15.1
OPN_3	Recommendations for future development	Refine formulary/list	33	13.8
OPN_3	Recommendations for future development	Policy support	32	13.4
OPN_3	Recommendations for future development	Education and awareness	32	13.4
OPN_3	Recommendations for future development	Risk assessment mechanisms	29	12.1
OPN_3	Recommendations for future development	Tiered authorization	24	10
OPN_3	Recommendations for future development	Lack of legal safeguards	12	5

**Figure 7:**
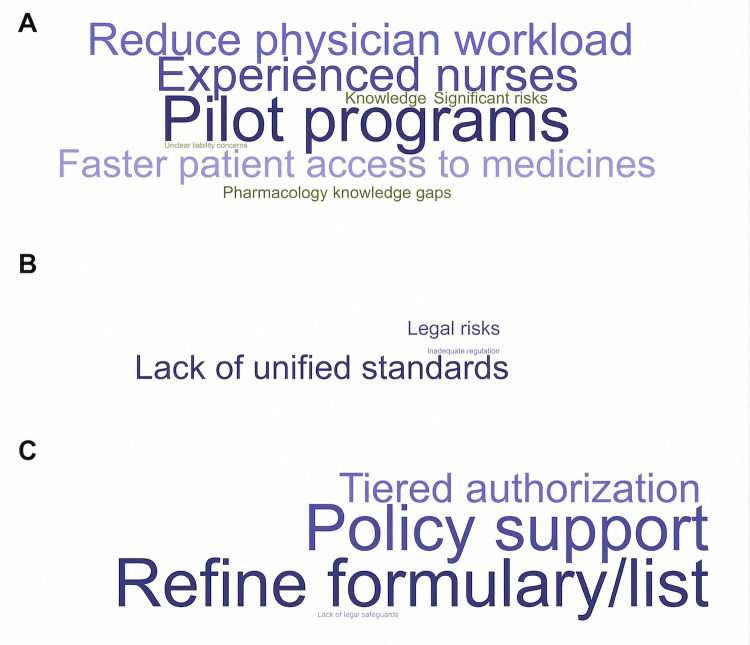
Word cloud analysis of open-ended question responses. **(A)** The greatest barriers to implementing nurse prescribing (OPN1); **(B)** Key measures to ensure safe implementation (OPN2); **(C)** Recommendations for future development (OPN3). Each word cloud visualizes high-frequency keywords extracted from responses to three open-ended questions. Font size is proportional to word frequency, indicating the relative prominence of each term in participants’ responses.

In responses to “the greatest barriers to implementing nurse prescribing” (OPN1) (Figure 7A), high-frequency terms such as “scope definition,” “training standards,” “pilot programs,” and “nursing experience” indicated that participants widely regarded unclear institutional frameworks and insufficient competency standards as major barriers. This theme highlighted practical concerns related to professional qualifications, prescribing boundaries, and role accountability.

For the question on “key measures to ensure safe implementation” (OPN_2_) (Figure 7B), keywords such as “physician-supervised prescribing,” “tiered prescription privileges,” “drug traceability,” and “legal risks” revealed strong concerns about regulatory oversight and legal safeguards. Respondents emphasized that implementation should be based on a system that ensures safety, accountability, and legal clarity.

In “recommendations for future development” (OPN3) (Figure 7C), frequent terms such as “policy support,” “refined drug formularies,” “risk assessment mechanisms,” and “legal protection” reflected a shared expectation for policy endorsement, institutional refinement, and tiered authorization. Overall, respondents expressed a clear desire to advance the standardization and sustainability of nurse prescribing through supportive policies and system-level innovation.

Taken together, the open-ended responses revealed a progressive logic in the development of nurse prescribing: from addressing institutional constraints, to establishing standardized governance, and ultimately to promoting long-term development. Word-frequency statistics and word-cloud analysis consistently highlighted participants' concerns regarding safety, standardization, and policy support. These qualitative themes were integrated with the quantitative findings through a joint display matrix (Table 7). The combined evidence showed convergence between stronger support among nurses, shared support for low-risk prescribing, and qualitative emphasis on scope definition, competency-based training, and legal safeguards. The integrated interpretation therefore supports a phased and safety-oriented implementation pathway.

**Table 7:** Joint display integrating quantitative findings and qualitative themes related to pediatric nurse prescribing.

Quantitative finding	Qualitative theme	Integrated interpretation
Nurses reported higher overall support than physicians for prescriptive authority.	Professional role expansion was viewed positively, but respondents emphasized the need for clear boundaries and accountability.	Attitudinal differences reflect different professional positions, but implementation may be advanced when role boundaries are explicitly defined.
Both groups showed similar support for basic and low-risk prescribing categories.	High-frequency terms highlighted scope definition, tiered authorization, and basic formularies.	Consensus is greatest for limited, low-risk prescribing, supporting a phased introduction strategy.
Perceived benefits and training needs were positive predictors of support.	Respondents repeatedly called for standardized training, supervised practice, and competency assessment.	Implementation readiness depends not only on perceived value, but also on a credible training and certification pathway.

## Discussion

This study compared pediatric nurses and physicians on support for nurse prescribing, identified factors associated with that support, and integrated quantitative findings with qualitative implementation concerns. Compared with previous Chinese studies that have mainly described general attitudes toward role expansion, this study contributes specialty-specific evidence from pediatric settings and shows how attitudinal differences coexist with practical consensus on low-risk prescribing. The finding that nurses expressed stronger support than physicians is broadly consistent with international evidence from settings where nurse prescribing has already been implemented (1, 3, 4). The pediatric focus is important because this specialty involves heavy workload, complex caregiver communication, and rapid clinical response, all of which make workflow-sensitive role design particularly relevant.

Perceived benefits and training needs emerged as the main drivers of support for nurse prescribing. This mechanism-level finding aligns with prior policy research suggesting that support is stronger when clinicians perceive clear service gains and a credible pathway to competence (16, 17). In practical terms, training readiness should be interpreted not only as recognition of competence gaps but also as willingness to participate in role transition. For curriculum development, these findings support training packages that combine pediatric pharmacology, dosage calculation, legal-regulatory content, prescription review, and supervised clinical practice. Framing nurse prescribing as a competency-based implementation process rather than a simple transfer of authority may therefore improve acceptability and safety.

Physicians showed lower support for nurse prescribing, reflecting stronger concerns about professional boundaries, accountability, and safety. From a professional-boundary perspective, this pattern can be understood as role negotiation during anticipated task redistribution rather than simple resistance to change. Importantly, however, the two groups did not differ substantially in their support for basic and commonly used medications, suggesting that consensus may be achievable when scope, accountability, and escalation rules are clearly defined. Institutional design should therefore prioritize explicit drug classification, tiered authorization, and physician-nurse collaborative mechanisms to reduce role ambiguity and strengthen trust. These implications may also be relevant to other countries that are considering phased or specialty-specific nurse prescribing models in legally restrictive settings.

Methodologically, this study used a convergent mixed-methods design and a joint display matrix to connect statistical patterns with frontline implementation concerns. This approach adds to the implementation science literature by showing not only whether stakeholders differ, but also which practical conditions appear necessary for adoption. The qualitative findings indicated that concerns about nurse prescribing were concentrated on structural issues, including unclear scope, lack of standardized training, and limited pilot experience, while suggested solutions focused on supervised prescribing, electronic prescription review, and legal oversight. These findings help explain why support was strongest for low-risk prescribing scenarios and more cautious for broader authority.

Risk perception showed a negative trend in its association with support among nurses. Although this relationship did not reach statistical significance, it suggests that enthusiasm for role expansion may weaken when safety systems are perceived as insufficient. This pattern reinforces the need to institutionalize risk boundaries through measures such as electronic prescription review, physician-nurse co-signature for selected scenarios, and tiered authorization linked to training and case complexity. In implementation terms, these safeguards can function as enabling conditions rather than merely restrictive controls.

Despite the differences in overall attitudes, the two groups converged on technical issues such as prescribing scope, training pathways, and safety measures, suggesting substantial potential for policy-level consensus. Nurse prescribing should therefore be framed not as a zero-sum transfer of authority, but as a staged reform that aligns interests through limited authorization under institutional safeguards. A practical pathway would be to initiate pilot programs in high-frequency settings such as pediatrics and chronic disease management, beginning with a restricted formulary of low-risk medications. Such pilots could generate implementation evidence, build interprofessional trust, and inform broader expansion across specialized nursing fields.

To our knowledge, this study is among the first to focus specifically on pediatric settings when comparing nurses’ and physicians' views on nurse prescribing in China. By integrating quantitative analysis with text mining and thematic analysis, it provides both empirical evidence on stakeholder support and practical insight into implementation conditions. Several limitations should be acknowledged. Convenience sampling and voluntary participation may have introduced selection bias and social desirability bias, and the anonymous design prevented comparison between respondents and non-respondents. The sample was limited to tertiary hospitals in one city, which may restrict generalizability. Although the final sample exceeded the prespecified target and was adequate for the regression analyses performed, no longitudinal follow-up or policy intervention evaluation was conducted. In addition, although internal consistency and preliminary structural adequacy were assessed, full exploratory or confirmatory factor analysis was not performed. The binary operationalization of support may also have reduced attitudinal nuance by collapsing neutral and negative responses into a single category, and some predictors may have been conceptually related to the support outcome. Finally, interpretation of the open-ended responses may have been influenced by the researchers' disciplinary backgrounds and prior assumptions, although thematic decisions were reviewed within the research team. Future studies should extend sampling to more diverse regions and specialties, test alternative outcome specifications, and incorporate pilot-based evaluations of implementation, patient outcomes, and physician-nurse collaboration.

## Conclusion

This study showed that pediatric nurses and physicians differed in their overall support for nurse prescribing, but shared substantial agreement on low-risk prescribing, training needs, and safety conditions. The findings support a phased and safety-oriented implementation pathway centered on restricted scope, competency-based training, and clear regulatory safeguards. Future research should evaluate pilot programs across more diverse settings and examine patient-level outcomes of implementation.

## Data Availability

The original contributions presented in the study are included in the article/Supplementary Material, further inquiries can be directed to the corresponding author/s.
