# Supplementary material for: Multidisciplinary perspectives on pediatric nurse prescribing: a mixed-methods analysis of attitudes and consensus
Source: Front Pediatr. 2026 May 20;14:1747318. doi: 10.3389/fped.2026.1747318 (PMC13229851; doi:10.3389/fped.2026.1747318)
Supplement: Supplementary file 1 [file Table1.docx]

**Table S1. Questionnaire Items and Variable Coding Scheme.**

| **Variable** | **Description (CN/EN)** | **Module** | **Type** | **Value/Coding** |
| --- | --- | --- | --- | --- |
| ID | Sample ID | Metadata | String | Free input (recommended unique) |
| DATE | Date of completion | Metadata | Date | YYYY-MM-DD |
| DUR_SEC | Response duration (seconds) | Metadata | Numeric | Non-negative integer |
| VALID | Validity flag | Metadata | Numeric | 1=valid, 0=invalid |
| DEM_1 | Occupational role | Demographics | Numeric | 1=Nurse, 2=Physician |
| DEM_2 | Gender | Demographics | Numeric | 1=Male, 2=Female |
| DEM_3 | Age (years) | Demographics | Numeric | Integer |
| DEM_4 | Highest education level | Demographics | Numeric | 1=Secondary vocational, 2=Associate, 3=Bachelor or above |
| DEM_5 | Professional title | Demographics | Numeric | 1=Junior, 2=Intermediate, 3=Senior |
| DEM_6 | Years of experience | Demographics | Numeric | Integer |
| DEM_7 | Hospital level | Demographics | Numeric | 1=Tertiary A, 2=Secondary, 3=Community/Township/Pediatric specialty |
| DEM_8 | Department type | Demographics | Numeric | 1=Outpatient, 2=Inpatient, 3=Emergency, 4=Other |
| ATT_1 | Overall support for nurse prescribing | General Attitude | Likert | 1=Strongly disagree … 5=Strongly agree |
| ATT_2 | Prescribing helps improve efficiency | General Attitude | Likert | 1…5 |
| ATT_3 | Prescribing helps reduce physician workload | General Attitude | Likert | 1…5 |
| ATT_4 | Prescribing enhances the status of nursing profession | General Attitude | Likert | 1…5 |
| REQ_1 | Minimum education requirement | Prescribing Eligibility | Numeric | 1=Secondary vocational, 2=Associate, 3=Bachelor or above |
| REQ_2 | Minimum title requirement | Prescribing Eligibility | Numeric | 1=Junior, 2=Intermediate, 3=Senior |
| REQ_3 | Minimum years of experience | Prescribing Eligibility | Numeric | 1=<5 years, 2=5-10 years, 3=>10 years |
| REQ_4 | Qualification requires unified training and exam | Prescribing Eligibility | Likert | 1…5 |
| CAT_1 | Support for disinfectants/antimicrobial agents | Prescribing Scope Support | Likert | 1…5 |
| CAT_2 | Support for solution-based drugs | Prescribing Scope Support | Likert | 1…5 |
| CAT_3 | Support for respiratory system drugs | Prescribing Scope Support | Likert | 1…5 |
| CAT_4 | Support for influenza-related medications | Prescribing Scope Support | Likert | 1…5 |
| CAT_5 | Support for anti-diarrheal/laxative drugs | Prescribing Scope Support | Likert | 1…5 |
| CAT_6 | Support for dermatological/allergic condition drugs | Prescribing Scope Support | Likert | 1…5 |
| CAT_7 | Support for nebulization and respiratory therapy devices | Prescribing Scope Support | Likert | 1…5 |
| CAT_8 | Support for pressure injury wound dressings | Prescribing Scope Support | Likert | 1…5 |
| CAT_9 | Support for infusion and injection apparatus | Prescribing Scope Support | Likert | 1…5 |
| CAT_10 | Support for wound/stoma/urinary supplies | Prescribing Scope Support | Likert | 1…5 |
| CAT_11 | Support for nutritional therapy drugs | Prescribing Scope Support | Likert | 1…5 |
| CAT_12 | Support for emergency medications | Prescribing Scope Support | Likert | 1…5 |
| BEN_1 | Improves pediatric patient access | Perceived Benefits | Likert | 1…5 |
| BEN_2 | Reduces physician workload | Perceived Benefits | Likert | 1…5 |
| BEN_3 | Enables faster initiation of treatment | Perceived Benefits | Likert | 1…5 |
| BEN_4 | Reduces medical resource wastage | Perceived Benefits | Likert | 1…5 |
| BEN_5 | Enables individualized care | Perceived Benefits | Likert | 1…5 |
| BEN_6 | Enhances nursing motivation and efficiency | Perceived Benefits | Likert | 1…5 |
| BEN_7 | Promotes harmonious nurse-physician-patient relationships | Perceived Benefits | Likert | 1…5 |
| BEN_8 | Improves service quality and competitiveness | Perceived Benefits | Likert | 1…5 |
| BEN_9 | Drives healthcare reform and innovation | Perceived Benefits | Likert | 1…5 |
| RISK_1 | Risk of medication misuse/adverse reactions | Risk Perception | Likert | 1…5 |
| RISK_2 | Risk of misdiagnosis | Risk Perception | Likert | 1…5 |
| RISK_3 | Risk of delayed treatment due to diagnostic errors | Risk Perception | Likert | 1…5 |
| RISK_4 | Risk of overtreatment/resource waste | Risk Perception | Likert | 1…5 |
| RISK_5 | Suboptimal treatment outcomes | Risk Perception | Likert | 1…5 |
| RISK_6 | Blurred professional boundaries/legal risk | Risk Perception | Likert | 1…5 |
| COLL_1 | Physicians generally accept nurse prescribing | Institutional Trust/Collaboration | Likert | 1…5 |
| COLL_2 | Hospital systems ensure legality/compliance | Institutional Trust/Collaboration | Likert | 1…5 |
| COLL_3 | Need for physician review mechanism | Institutional Trust/Collaboration | Likert | 1…5 |
| COLL_4 | Interprofessional communication ensures safe prescribing | Institutional Trust/Collaboration | Likert | 1…5 |
| TRN_1 | Basic pharmacology knowledge (self-assessed) | Training and Competency | Likert | 1…5 |
| TRN_2 | Ability to assess indications for common pediatric drugs (self-assessed) | Training and Competency | Likert | 1…5 |
| TRN_3 | Willingness to attend prescribing-related training | Training and Competency | Likert | 1…5 |
| TRN_4 | Current nursing education meets prescribing requirements | Training and Competency | Likert | 1…5 |
| TRN_5 | Confidence in professional ability to prescribe | Training and Competency | Likert | 1…5 |
| OPN_1 | Primary barriers to nurse prescribing (open text) | Open-ended | Text | — |
| OPN_2 | Key measures for safe implementation (open text) | Open-ended | Text | — |
| OPN_3 | Recommendations for future development (open text) | Open-ended | Text | — |
