# Supplementary material for: Multidisciplinary perspectives on pediatric nurse prescribing: a mixed-methods analysis of attitudes and consensus
Source: Front Pediatr. 2026 May 20;14:1747318. doi: 10.3389/fped.2026.1747318 (PMC13229851; doi:10.3389/fped.2026.1747318)
Supplement: Supplementary file 2 [file Table2.docx]

**Pediatric Nurses' Perceptions and Attitudes Toward Prescriptive Authority: Survey (Research Version)**

**Instructions for Completion**

This questionnaire aims to assess pediatric nurses' and physicians' perceptions, attitudes, and influencing factors related to nurse prescriptive authority.

All responses are confidential and used solely for academic research purposes.

**How to respond:**

For single-choice questions, mark "√" next to the appropriate option.

For Likert-scale items, select a score from 1 to 5 based on your level of agreement (1 = Strongly Disagree; 5 = Strongly Agree).

**Section I. Demographic and Professional Information**

1. Gender: □ Male □ Female
2. Age: ____ years
3. Education: □ Secondary nursing diploma □ Associate degree □ Bachelor's degree or above
4. Professional title: □ Junior □ Intermediate □ Senior
5. Years of experience: □ <3 years □ 3–5 years □ 5–10 years □ >10 years
6. Hospital level: □ Tertiary A □ Secondary □ Community/Township Health Center
7. Professional role: □ Pediatric nurse □ Pediatric physician
8. Department type: □ Outpatient □ Inpatient □ Emergency □ Other: __________

**Section II. Overall Attitudes Toward Nurse Prescriptive Authority**

(1 = Strongly Disagree; 5 = Strongly Agree)

1. I support granting prescriptive authority to pediatric nurses. 1□ 2□ 3□ 4□ 5□
2. Nurse prescriptive authority improves healthcare service efficiency. 1□ 2□ 3□ 4□ 5□
3. Nurse prescriptive authority reduces physicians' workload. 1□ 2□ 3□ 4□ 5□
4. Nurse prescriptive authority enhances the professional status of nursing. 1□ 2□ 3□ 4□ 5□

**Section III. Eligibility Criteria for Nurse Prescriptive Authority**

1. What is the minimum education level required for nurses to apply for prescriptive authority?

□ Secondary nursing diploma □ Associate degree □ Bachelor's degree or above

1. What is the minimum professional title required?

□ Junior □ Intermediate □ Senior

1. What is the minimum work experience required?

□ <5 years □ 5–10 years □ >10 years

1. Nurses should obtain prescriptive authority only after standardized training and examination.

1□ 2□ 3□ 4□ 5□

**Section IV. Support for Scope of Nurse Prescribing (corresponding to Table 3)**

(1 = Strongly Oppose; 5 = Strongly Support)

1. Antiseptics and disinfectants (e.g., ethanol, chlorhexidine) 1□ 2□ 3□ 4□ 5□
2. Intravenous solutions (e.g., saline, glucose injections) 1□ 2□ 3□ 4□ 5□
3. Respiratory medications (e.g., aminophylline, budesonide, cephalosporins) 1□ 2□ 3□ 4□ 5□
4. Influenza-related drugs (e.g., antivirals, antipyretics, antitussives) 1□ 2□ 3□ 4□ 5□
5. Gastrointestinal medications (e.g., montmorillonite, probiotics) 1□ 2□ 3□ 4□ 5□
6. Dermatological and allergy medications (e.g., miconazole, promethazine) 1□ 2□ 3□ 4□ 5□
7. Nebulization and respiratory therapy equipment 1□ 2□ 3□ 4□ 5□
8. Pressure ulcer and wound dressing supplies 1□ 2□ 3□ 4□ 5□
9. Infusion and injection devices 1□ 2□ 3□ 4□ 5□
10. Ostomy and catheter-related nursing products 1□ 2□ 3□ 4□ 5□
11. Nutritional support medications 1□ 2□ 3□ 4□ 5□
12. Emergency drugs (e.g., epinephrine, dopamine) 1□ 2□ 3□ 4□ 5□

**Section V. Perceived Benefits of Nurse Prescriptive Authority**

(1 = Strongly Disagree; 5 = Strongly Agree)

1. Increases convenience for pediatric patients seeking care 1□ 2□ 3□ 4□ 5□
2. Reduces physician workload 1□ 2□ 3□ 4□ 5□
3. Improves treatment efficiency for pediatric patients 1□ 2□ 3□ 4□ 5□
4. Minimizes waste of healthcare resources 1□ 2□ 3□ 4□ 5□
5. Enhances personalized care and treatment 1□ 2□ 3□ 4□ 5□
6. Boosts nurses' professional engagement 1□ 2□ 3□ 4□ 5□
7. Improves nurse–patient relationships and care quality 1□ 2□ 3□ 4□ 5□
8. Enhances hospital service quality and competitiveness 1□ 2□ 3□ 4□ 5□
9. Promotes healthcare system reform 1□ 2□ 3□ 4□ 5□

**Section VI. Perceived Risks of Nurse Prescriptive Authority**

(1 = Strongly Disagree; 5 = Strongly Agree)

1. May increase the risk of medication misuse and adverse reactions 1□ 2□ 3□ 4□ 5□
2. May lead to misdiagnosis or diagnostic errors 1□ 2□ 3□ 4□ 5□
3. May result in overtreatment 1□ 2□ 3□ 4□ 5□
4. May compromise treatment outcomes 1□ 2□ 3□ 4□ 5□
5. May increase legal risks and liability stress 1□ 2□ 3□ 4□ 5□
6. May blur professional role boundaries 1□ 2□ 3□ 4□ 5□

**Section VII. Institutional Trust and Collaborative Mechanisms**

(1 = Strongly Disagree; 5 = Strongly Agree)

1. Physicians generally accept nurses having prescriptive authority 1□ 2□ 3□ 4□ 5□
2. Hospitals can provide adequate institutional safeguards 1□ 2□ 3□ 4□ 5□
3. Nurse prescriptions should be subject to physician review 1□ 2□ 3□ 4□ 5□
4. Effective nurse–physician communication ensures prescription safety 1□ 2□ 3□ 4□ 5□

**Section VIII. Training and Self-assessed Competence**

(1 = Strongly Disagree; 5 = Strongly Agree)

1. I possess basic pharmacological knowledge 1□ 2□ 3□ 4□ 5□
2. I am capable of assessing common pediatric medication indications 1□ 2□ 3□ 4□ 5□
3. I am willing to participate in prescriptive authority training 1□ 2□ 3□ 4□ 5□
4. I believe current nursing education prepares me for prescriptive authority 1□ 2□ 3□ 4□ 5□
5. I feel confident in my ability to exercise prescriptive authority 1□ 2□ 3□ 4□ 5□

**Section IX. Policy and Implementation Suggestions**

1. In which type of institution should nurse prescriptive authority be piloted?

□ Tertiary A hospital □ Secondary hospital □ Community Health Center □ Pediatric Specialty Hospital □ Township Health Center

1. What forms of oversight should nurse prescribing include?

□ Physician review □ System-based prescription auditing □ Periodic evaluation □ Legal supervision

1. Which institutions should be responsible for training?

□ National Health Commission □ Nursing Association □ Hospitals □ Medical schools □ Other: __________

1. What categories of drugs should be included in nurse prescriptive authority?

□ Basic medications □ Antibiotics □ Chronic disease medications □ Nursing consumables □ Emergency drugs

**Section X. Open-Ended Responses**

1. In your opinion, what is the biggest obstacle to implementing nurse prescriptive authority?
2. What are the key measures to ensure the safe implementation of nurse prescriptive authority?
3. What are your suggestions for the future development of pediatric nurse prescriptive authority in China?

**Thank you for your participation and support!**
